# Event-related components are structurally represented by intrinsic event-related potentials

**DOI:** 10.1038/s41598-021-85235-0

**Published:** 2021-03-11

**Authors:** Chong-Chih Tsai, Wei-Kuang Liang

**Affiliations:** 1 Institute of Cognitive Neuroscience, National Central University, Taoyuan, Taiwan; 2 Cognitive Intelligence and Precision Healthcare Center, National Central University, Taoyuan, Taiwan; 3 Department of Psychiatry, Taoyuan Armed Forces General Hospital, Taoyuan, Taiwan

**Keywords:** Cognitive neuroscience, Electroencephalography - EEG

## Abstract

The detection of event-related potentials (ERPs) through electroencephalogram (EEG) analysis is a well-established method for understanding brain functions during a cognitive process. To increase the signal-to-noise ratio (SNR) and stationarity of the data, ERPs are often filtered to a wideband frequency range, such as 0.05–30 Hz. Alternatively, a natural-filtering procedure can be performed through empirical mode decomposition (EMD), which yields intrinsic mode functions (IMFs) for each trial of the EEG data, followed by averaging over trials to generate the event-related modes. However, although the EMD-based filtering procedure has advantages such as a high SNR, suitable waveform shape, and high statistical power, one fundamental drawback of the procedure is that it requires the selection of an IMF (or a partial sum of a range of IMFs) to determine an ERP component effectively. Therefore, in this study, we propose an *intrinsic* ERP (*i*ERP) method to overcome the drawbacks and retain the advantages of event-related mode analysis for investigating ERP components. The *i*ERP method can reveal multiple ERP components at their characteristic time scales and suitably cluster statistical effects among modes by using a tailored definition of each mode’s neighbors. We validated the *i*ERP method by using realistic EEG data sets acquired from a face perception task and visual working memory task. By using these two data sets, we demonstrated how to apply the *i*ERP method to a cognitive task and incorporate existing cluster-based tests into *i*ERP analysis. Moreover, *i*ERP analysis revealed the statistical effects between (or among) experimental conditions more effectively than the conventional ERP method did.

## Introduction

Event-related potential (ERP) analysis has been employed for decades to investigate the neural mechanisms of sensory, motor, and cognitive processes through electroencephalograms (EEGs). This analysis was conducted using the average of the time-locked EEG response (in general, for an event or stimulus) over trials with a certain experimental condition for each participant. A conventional practice for increasing the signal-to-noise ratio (SNR) and stationarity of the EEG data before or after the averaging process involves applying a filter (or filters) to the data. Typically, a wideband filter, such as a 0.05–30-Hz filter (which can be achieved using an online 0.05–100-Hz bandpass filter during EEG acquisition and an offline 30-Hz low-pass filter in ERP analysis) is recommended to avoid the potential distortion of ERP waveforms^1^. Conventionally, the filtering procedure employed in ERP analysis is performed using Fourier-based filters, such as finite impulse response (FIR), infinite impulse response (IIR), and wavelet filters.

In the past decade, an alternative filtering process achieved through empirical mode decomposition (EMD)^2,3^ was introduced for analyzing ERPs^4,5^, which are referred to as event-related modes^6^. In the alternative process, EMD serves as a natural dyadic filter bank^7^ to decompose the EEG signals of each trial into a finite set of intrinsic mode functions (IMFs). An IMF is a function that satisfy two conditions: (1) the number of extrema (minima and maximum) and the number of zero-crossings must either be equal or differ at most by one; and (2) at any point, the mean value of the upper envelope (defined by all local maxima) and lower envelope (defined by all local minima) is zero. The EMD extracts IMFs by a sifting process which removes the local mean of the upper and lower envelopes iteratively (see “Materials and methods”). In order to perform the event-related mode analysis, IMFs with either the similar frequency distribution or the same order (if there exists high interchannel, intertrial, and intersubject frequency distribution consistency in the order of IMFs) in different trials are averaged to obtain the “mode”. Furthermore, the concept of the event-related mode has been extended to include the “composite mode” formed by taking the partial sum of two or more above-mentioned “pure modes” (e.g., those modes with frequency distribution corresponding to delta, theta, and alpha oscillations). In contrast to Fourier-based filters, this natural filtering strategy may help to give a more physically meaningful representation^2^ of an ERP component. This method has been used for studying ERP components involved in various cognitive functions, such as auditory perception, sentence comparison, olfactory perception, visual perception, and attentional control^5,6,8–10^. A 2016 study by Hsu et al.^11^ investigated the event-related mode of a partial sum of two consecutive IMFs obtained through ensemble EMD^12^ (EEMD) in a task of auditory perception. The event-related mode formed by the two IMFs, which had a frequency distribution of 2–8 Hz, had a considerably higher statistical power and required fewer trials for each condition than the conventional ERP did for investigating the mismatch negativity effect. A common feature of the aforementioned studies is that they utilized an IMF or IMFs obtained through EMD or EEMD to generate narrowband data while maintaining an undistorted waveform shape in a certain time range^13^ to represent their ERP components effectively. The success of these studies indicated that an ERP component might have a characteristic time scale and waveform. The concept of "characteristic time scale" was introduced in the basic assumption of EMD^2^, and was defined by the time lapse between the extrema (from a minimum to its following maximum, or vice versa) in a given IMF. Thus, with the appropriate selection of the IMF(s) corresponding to an ERP component’s characteristic time scale, the ERP component can be clearly assessed. However, whether the characteristic time scales of the critical ERP components are similar for a cognitive task (e.g., a working memory task) that comprises two or more critical ERP components must be determined. When researchers select a specific IMF or a partial sum of IMFs to better resolve one ERP component, the resolution of the other ERP components may be negatively affected if the characteristic time scales of the ERP components are different. However, further justification is required if a study must use different IMFs or partial sums of IMFs to investigate different ERP components. Considering that the number of IMFs is finite, to achieve the aim of revealing every critical ERP component in its corresponding characteristic time scale for a cognitive task, we propose using all possible IMFs and their various partial sums (i.e., all possible “modes” after averaging over trials) to represent ERP components. This goal is difficult to achieve with a conventional bandpass filter because an infinite number of combinations of lower and upper cutoff frequencies exist. In order to make our proposed representation applicable to practical EEG data analysis, the following two points should be considered. First, not all of the IMFs and partial sums are mathematically independent. Second, including all possible modes would add a new dimension to ERP representation [i.e., the “mode” dimension in addition to the one-dimensional (1D) time or two-dimensional (2D) channel × time dimensions in the conventional ERP method], which can result in a number of comparisons several times that of the conventional ERP representation [i.e., a more massive multiple comparison problem (MCP)].

To avoid the MCP when exploring ERPs, we propose a method in this study that involves averaging over trials for a plausible range of IMFs and their different partial sums as well as using tailored definitions of neighbors in different modes for clustering statistical effects. The features of the proposed method are as follows. First, instead of the EMD or EEMD methods used in previous studies, we employed an improved complete ensemble EMD with adaptive noise^14–16^ (CEEMDAN) method for identifying IMFs. The CEEMDAN method offers valuable improvements to the EEMD method. Compared with the EEMD method, the CEEMDAN method has a lower reconstruction error (i.e., the noise residual within IMFs) and higher consistency of frequency distribution in the order of IMFs for different noisy signals. The aforementioned characteristics are critical for the proposed method, which requires high consistency of the IMF order across channels, trials, and subjects for a specific frequency range of brain oscillation. The CEEMDAN method can also be combined with a complementary approach for generating white noise to reduce the final residual noise (see “Materials and methods”). Moreover, exploring the ERPs for all possible IMFs and their various partial sums enables the investigation of the ERP effect from the narrowest frequency band (i.e., each single IMF) to the widest natural frequency passband (i.e., the sum from a specified initial IMF to the final component of the CEEMDAN method). When the aforementioned approach is adopted, the statistical effect of a given ERP component becomes pronounced when at least one critical IMF is involved. This effect vanishes after exclusion of the critical IMF(s), revealing the characteristic time scale of the ERP component. Finally, the proposed method incorporates a procedure for clustering statistical effects from various IMFs and their partial sums (i.e., different modes) within a 2D [mode, time] or 3D [channel, mode, time] space. In this procedure, two similar modes are identified as neighboring modes if and only if exactly one element (one ordinal number of an IMF) is present in the set given by the exclusive OR operation between the two sets of *ordinal numbers* of the constituent IMFs of the two modes. With the aforementioned assignment of neighboring modes and use of the same neighbor identification in other dimension(s) (i.e., “time” in the 2D space or “channel and time” in the 3D space) as in conventional ERP, any existing cluster-based statistical method [e.g., the standard cluster-based nonparametric permutation (CBnPP) method^17^ and the threshold-free cluster enhancement (TFCE) method^18^] can be employed to cluster the statistical effects among various modes. Thus, multiple ERP components with various characteristic time scales can be obtained simultaneously. The proposed method is hereinafter referred to as the *intrinsic* ERP (*i*ERP) method because it involves using numerous IMFs. The averaged IMFs and partial sums of the IMFs are referred to as *i*ERP modes. The application of the *i*ERP method is demonstrated using realistic EEG data acquired from a face perception task and working memory task.

## Materials and methods

**Figure 1:**
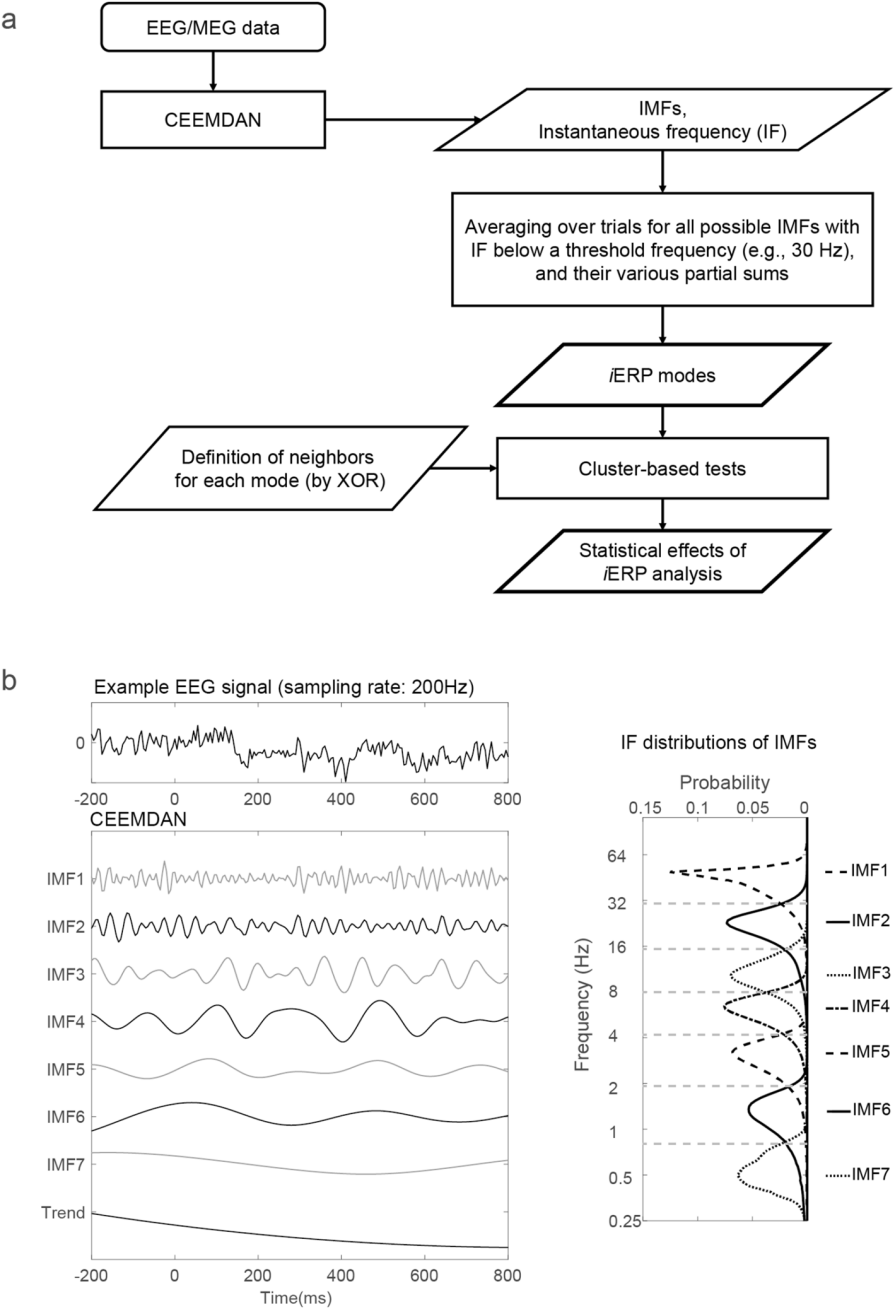
*i*ERP analysis of EEG/MEG signals. (**a**) Flowchart of *i*ERP analysis. The *i*ERP method involves using the improved CEEMDAN method to obtain IMFs for EEG/MEG data of each trial at each channel for each condition and participant. The IMFs and their various partial sums are then averaged over trials within each condition for each channel and each participant to form *i*ERP modes. The statistical effects were obtained by incorporating a special definition of neighbors for each mode in existing cluster-based statistical tests. (**b**) Illustration of the signal decomposition with the CEEMDAN method and IF distributions of the IMFs.

The *i*ERP method integrates (1) an improved CEEMDAN method, (2) a complementary approach for white noise generation, (3) a method for organizing *i*ERP modes, and (4) a method for clustering statistical effects. Each of the items (2–4) has a certain degree of novelty. A flowchart of the *i*ERP method is displayed in Fig. 1a. Each process in the aforementioned method is detailed in the following text.

### Introduction to EMD

The EMD method decomposes a series of signal into a generally small number of IMFs through the sifting process. The steps of EMD and its sifting process are described as follows:

1. Find all minima and maxima of the data *x*.
2. All the local maxima (minima) are connected by a cubic spline line to acquire the upper (lower) envelope first, followed by obtaining the first component *h* by taking the difference between the data *x* and the local mean of the upper and lower envelopes.
3. Treat *h* as the data and repeat steps 1 and 2 as many times as required to satisfy some stopping criterion. The final *h* is designated as *c*_*1*_, which is the first IMF from the data. Then separate *c*_*1*_ from the rest of the data by *x* *−* *c*_*1*_ = *r*_*1*._
4. Treat the residue *r*_*1*_ as the data and repeat steps 1–3 to acquire the second IMF *c*_*2*_ and its corresponding residue *r*_*2*_ (obtained by *r*_*1*_ – *c*_*2*_).
5. Continue step 4 by treating the residue *r*_*k*_ as the data (*k* ≥ 2) to retrieve the IMF *c*_*k*+*1*_ until the final residue *r*_*n*_ becomes a function from which no further IMFs can be obtained (i.e., no more than two extrema).

In the above EMD process, the data *x* is decomposed into IMFs, *c*_*j*_, i.e., $x = \sum\nolimits_{j = 1}^{n} {c_{j} + r_{n} }$, where *r*_*n*_ designates the residue of the data *x*, after *n* number of IMFs are extracted. In this study, all the processes of EMD (within the CEEMDAN method) employ a stopping criterion suggested by Wu and Huang^12,19^ that fixes the sifting number to 10. This stopping criterion would lead to EMD being a “nearly perfect” dyadic filter for white noise while keeping the upper and lower envelopes of IMFs almost symmetric to the zero line. This dyadic filtering property is especially critical for the current study because it would also lead to the total number of IMFs of the “noise-added” data being close to log_2_*N* (*N*: the number of total data points).

### Improved CEEMDAN method

The CEEMDAN method is characterized by its “adaptive noise”. In this method, each realization of white Gaussian noise (WGN) is decomposed through EMD to produce “noise IMFs.” These noise IMFs given by a number of realizations of WGN form a “complete” ensemble of noises and are added to the original signals and the subsequent residues in a consistent order to generate “ensemble mean” IMFs for the data. The original notations employed by the developers of the CEEMDAN method are as follows: *E*_*k*_(.) is the operator that provides the *k*th mode of EMD, and *w*^(*i*)^ is a realization of *N*(0, 1) WGN. The steps of the CEEMDAN algorithm are briefly described as follows:

1. Apply EMD to the *l* realizations of the adaptive-noise-containing data *x*^(*i*)^ = *x* + *β*_0_*E*_1_(*w*^(*i*)^) to obtain the first mode and its residue *r*_1_^(*i*)^ for each realization. The first mode for each realization is obtained by the sifting process, and its residue is the remaining signal once the first mode has been removed from the realization.
2. Calculate the first residue of CEEMDAN by averaging all the residues *r*_1_^(*i*)^ in the *l* realizations: $\tilde{r}_{1} = \langle {r_{1}^{(i)} } \rangle$. The first IMF is obtained as follows: $\tilde{d}_{1} = x - \tilde{r}_{1}$.
3. Perform EMD on the *l* realizations $\tilde{r}_{1} + { }\beta_{1} E_{2} (w^{(i)} )$ to acquire the first mode and its residue *r*_2_^(*i*)^ for each realization. Thus, the second residue of CEEMDAN can be estimated as follows: $\tilde{r}_{2} = \langle {r_{2}^{(i)} } \rangle$. The second IMF is then obtained as follows: $\tilde{d}_{2} = \tilde{r}_{1} - \tilde{r}_{2}$.
4. Similar to step 3, for *k* = 3, …, *K*, recursively perform EMD on the *l* realizations $\tilde{r}_{k - 1} + \beta_{k - 1} E_{k} (w^{(i)} )$ to obtain the first mode and its residue *r*_*k*_^(*i*)^ for each realization. Thus, the *k*th residue of CEEMDAN can be obtained as follows: $\tilde{r}_{k} = \langle {r_{k}^{(i)} } \rangle$. The *k*th IMF is then obtained as follows: $\tilde{d}_{k} = \tilde{r}_{k - 1} - \tilde{r}_{k}$.

The coefficient ${ }\beta_{k} = \varepsilon_{k} {\text{std}}(\tilde{r}_{k} )/{\text{std}}(E_{k + 1} (w^{(i)} ))$ is used for controlling the SNR. In the two examples in this study, $\varepsilon_{k}$ was set as 0.2.

### Complementary approach for white noise generation

The proposed method also involves using a complementary approach for generating adaptive noise. The principle of the adopted complementary approach is similar to that of the complementary EEMD method proposed by Yeh et al.^20^ except that a complementary pair of adaptive noises (i.e., IMFs of the WGNs) is used instead of a complementary pair of WGNs. Therefore, for each of the aforementioned steps of the CEEMDAN algorithm, when a realization $\tilde{r}_{k - 1} + { }\beta_{k - 1} E_{k} (w^{(i)} )$ was adopted, its complementary realization $\tilde{r}_{k - 1} - { }\beta_{k - 1} E_{k} (w^{(i)} )$ was also included in the analysis to further reduce the residual noise in IMFs and the required size of the noise ensemble.

### Forming *i*ERP modes

In the proposed method, *i*ERP modes are obtained by averaging each possible IMF (i.e., a *pure mode*) or partial sum of the IMFs (i.e., a *composite mode*) over trials within each condition for each channel and each participant. Optionally, IMFs whose instantaneous frequencies (IFs) are higher than a certain threshold (e.g., 30 Hz) can be excluded from the set of constituent IMFs during the generation of *i*ERP modes. We expressed *i*ERP modes according to the initial and final orders of the constituent IMFs of each mode. For example, the IF distributions of the IMFs of the EEG data displayed in Fig. 1b indicate that oscillatory activity below the specified frequency of 30 Hz originated from the second and the following order of IMFs. Thus, the organization of *i*ERP modes can be expressed as *p*:*q*, where *p* and *q* = 2, …, *N*, with *p* <  = *q*. If *p* = *q*, the expression *p*:*p* denotes a *pure mode* obtained by averaging the *p*th IMF over trials, otherwise the expression *p*:*q* (*p* < *q*) designates a *composite mode* formed by averaging over trials the partial sum from the *p*th IMF to the *q*th IMF. In practical application, IMFs with IFs below the delta frequency range can be summed to form a single component. For example, the IMFs in Fig. 1b with an order equal to or higher than 6 have IFs lower than 2 Hz; thus, these IMFs can be summed to produce a single component. The summed single component is denoted by the order of its initial IMF of the sum, together with the suffix “+” (i.e., “6+” in the aforementioned example, signifying the component is obtained by summing from the sixth IMF to the “trend”). Therefore, “2:6+” indicates that the range of the partial sum is not only from the 2nd to the 6th IMF but also includes all IMFs following the 6th IMF. Thus, this 2:6+ partial sum yields a wideband *i*ERP mode that is similar to the conventional ERP with a 32 Hz lowpass.

### Clustering statistical effects

To determine the experimental effects and overcome the MCP in the *i*ERP method, we included a special definition of neighbors for the *i*ERP modes in the proposed method. This neighbor definition for the *i*ERP modes can be primarily used in cluster-based statistical analysis methods such as the CBnPP^17^ and TFCE method^18^ to cluster the statistical effects of various *i*ERP modes on the basis of 2D [mode, time] or 3D [channel, mode, time] adjacency. In the “mode” dimension, the neighbors of a given *i*ERP mode (denoted by ***a***) are identified using the following criterion: if the resulting set of the exclusive OR (XOR) operation between the set of ordinal numbers of constituent IMFs of any *i*ERP mode (denoted by ***b***) and the set of ordinal numbers of constituent IMFs of the *i*ERP mode ***a*** consists of only one element (i.e., an ordinal number of an IMF), then mode ***b*** is a neighbor of mode ***a***. For instance, the *i*ERP modes 2:5 and 3:5 are mutual neighbors because the XOR between the two corresponding sets of ordinal numbers of constituent IMFs only yields a set of one element (i.e., “2”); however, modes 2:5 and 3:6 are not mutual neighbors because the XOR between the two corresponding sets of ordinal numbers of constituent IMFs gives a set of two elements (i.e., [2, 6]). For the other dimension(s) (i.e., time in the 2D *i*ERP space or “channel and time” in the 3D *i*ERP space), the principle of neighbor identification remains the same as that in conventional ERP analysis. Specifically, two channels are considered neighbors if their distance is less than a threshold value (e.g., 40 mm), and two time bins are considered neighbors if they are adjacent to each other. Consequently, in any representation of *i*ERP (i.e., in the 2D [mode, time] or 3D [channel, mode, time] *i*ERP space), two data points are identified as “2- or 3-dimensional” neighbors if the two data points are mutual neighbors in only one dimension (e.g., “mode”) while sharing the same coordinate(s) in the other dimension(s) (e.g., [channel, time]).

The standard CBnPP method employed in the two examples in this study is a strategy for performing multiple comparisons while maintaining reasonable statistical power. We use the CBnPP test to reveal the differences of *i*ERP between two conditions, and to evaluate the correlations between the *i*ERP and participants’ behavioral data (e.g., working memory capacity). Based on the aforementioned 2- or 3-dimensional neighbor relationship, the CBnPP test for *i*ERP is described as follows:

1. For every data point in the *i*ERP space, perform a classical parametric statistical test to obtain the corresponding statistical value (e.g., *t* value for *t*-test, and correlation coefficient *ρ* for correlation analysis).
2. Select all data points whose statistical value is higher than some uncorrected threshold based on the sampling distribution of the statistical value under the null hypothesis (e.g., 97.5th quantile of a *T*-distribution, which is corresponding to a critical value in a two-tailed *t*-test at alpha-level 0.05).
3. Cluster the selected data points in *connected sets* on the basis of the 2- or 3-dimensional neighbor relationship.
4. Calculate the cluster-level *observed test statistic* by taking the sum of the statistical values obtained in step 2 within each cluster.
5. Generates the randomized data by a large number (e.g., 5000 times) of permutations on the variable of the parametric test statistic adopted in step 1 (e.g., participants’ conditions in a dependent samples *t*-statistic, or participants’ order in a correlation analysis).
6. For each permutation, repeat steps 1–3 for the randomized data and calculate the cluster-level test statistics by taking the sum of the statistical values within each randomly occurring cluster. Only the maximum of the cluster-level test statistics in each permutation is chosen for constructing the permutation distribution of the cluster-level *random test statistics*.
7. Finally, the cluster-level observed test statistics (i.e., without permutation) are further tested against the cluster-level random test statistics (i.e., the permutation distribution) under a prespecified critical alpha-level (e.g., α = 0.05, two-sided).

The TFCE method used in an example in this study is an extension of the standard CBnPP test. In the CBnPP test, the choice of the initial cluster-forming threshold is arbitrary, but its exact chosen value can have a non-negligible effect on the results. The advantage of the TFCE method is that it can keep the sensitivity benefits of the standard CBnPP test, while avoiding (or, minimizing) the arbitrary thresholding problem. Furthermore, various statistical tests have been implemented based on the TFCE approach, including those tests that are not available in the standard CBnPP, such as the TFCE version within-subjects and between-subjects one-way analysis of variance (ANOVA) and mixed-model two-way ANOVA. When any TFCE-based statistical test is applied to the *i*ERP data, the only requirement is to clearly define the 2- or 3-dimensional neighbor relationship in the 2D [mode, time] or 3D [channel, mode, time] *i*ERP space as we have mentioned above, without modifying its original algorithm^18^.

In this study, the codes for demonstrating the cluster-based statistical tests in the *i*ERP method are adapted from MATLAB functions in the Mass Univariate ERP toolbox^21^ and the ept_TFCE toolbox^22^ (https://github.com/Mensen/ept_TFCE-matlab) for the standard CBnPP and the TFCE method, respectively.

## Examples

To illustrate the application of the *i*ERP method to real data, we considered EEG data acquired from a face perception task and working memory task. The conventional ERP method was also applied with the aforementioned data to compare its performance with that of the proposed method.

Because of the long history of the application of ERPs in cognitive neuroscience, various a priori hypotheses have been introduced into conventional ERP analyses, e.g., an a priori time window or EEG sensor(s) for a certain ERP component. Although we suggest performing the *i*ERP analysis *without* these a priori hypotheses (i.e., in the 3D [channel, mode, time] space), in the following two examples, we will also demonstrate how to conduct the *i*ERP analysis based on established a priori knowledge to increase the flexibility in using the *i*ERP method. Once an a priori knowledge (i.e., a prespecified EEG sensor or time window) is incorporated, the *i*ERP analysis including its adopted cluster-based approach will be in a dimensionality-reduced space (i.e., a 2D [mode, time] space for an a priori sensor, or 2D [channel, mode] space for an a priori time window).

### Application of the *i*ERP method with the data acquired from a face perception task

**Figure 2:**
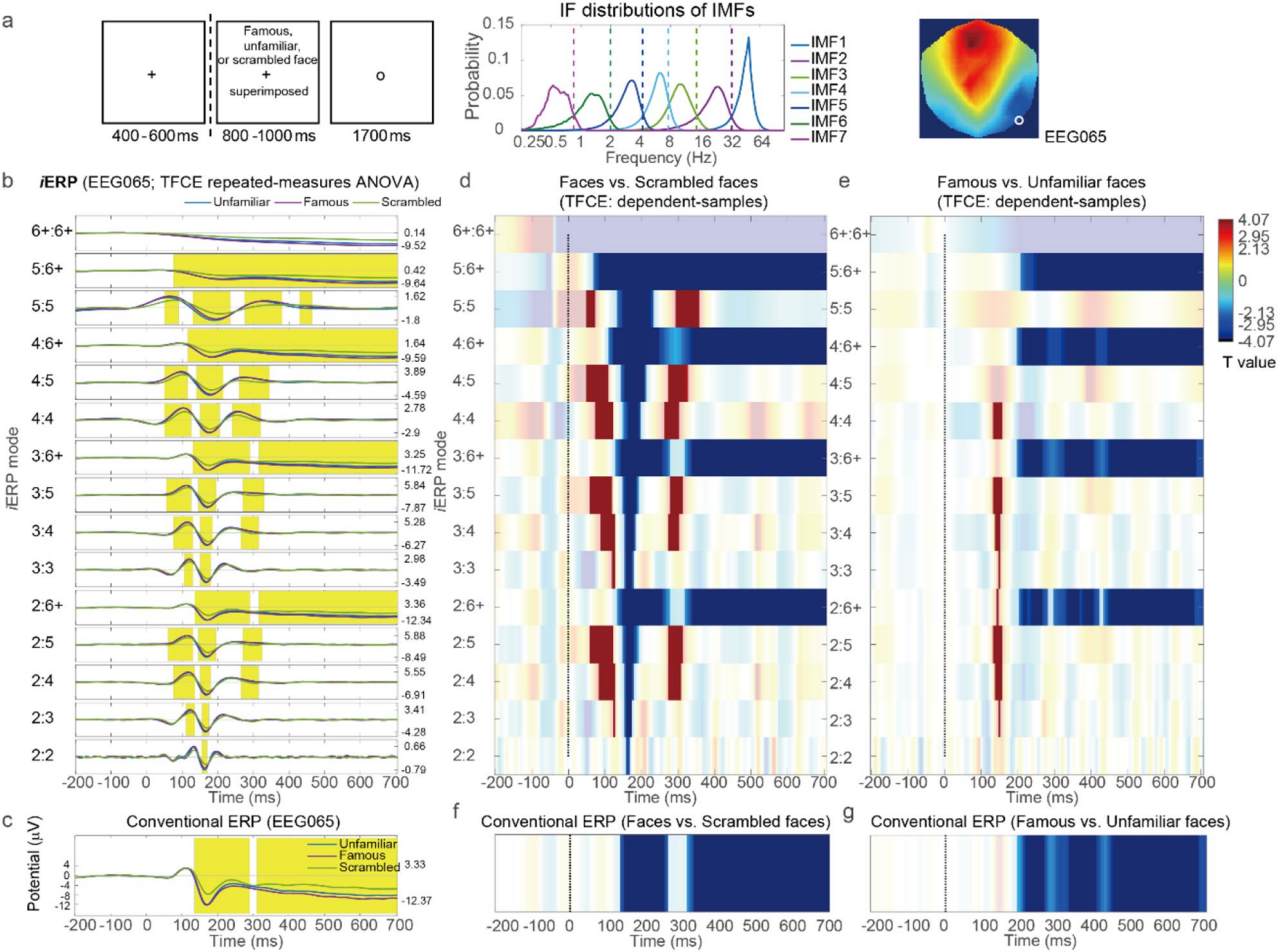
*i*ERP analysis on EEG data of the face perception task. (**a**) Illustration of the face perception task in the first example, the IF distributions of the IMFs of the 200-Hz EEG data, and the representative right temporoparietal EEG channel (EEG065). (**b**) *i*ERP results for the EEG signals from the EEG065 channel and TFCE repeated-measures ANOVA of the three conditions within the 2D [mode, time (50–700 ms)] domain. Time windows in yellow indicate *P* < 0.005 for TFCE. (**c**) Conventional ERP results corresponding to the *i*ERP results in (**b**). (**d**) Statistical contrast of the *i*ERPs between unscrambled faces (famous + unfamiliar) and scrambled faces (indicated by *T* values). Regions with completely opaque colors indicate *P* < 0.025 for TFCE. (**e**) Statistical contrast of the *i*ERP results between famous and unfamiliar faces. Conventions are the same as those in (**d**). (**f**) Conventional ERP contrast between unscrambled and scrambled faces. (**g**) Conventional ERP contrast between famous and unfamiliar faces.

The *i*ERP method was first applied to EEG data obtained from a publicly accessible multimodal data set (https://openneuro.org/datasets/ds000117/). These data were collected from 16 participants during a face perception task with multiple trials of famous, unfamiliar, and scrambled faces (Fig. 2a, left)^23,24^. The preprocessing procedure was the same as that described in chapter 42 of the SPM12 manual (https://www.fil.ion.ucl.ac.uk/spm/doc/manual.pdf). Thus, the downsampled EEG data set comprised trials with a sampling rate of 200 Hz. Research conducted using the conventional ERP method has indicated a greater negative deflection in N170 amplitude for unscrambled faces than for scrambled faces in right temporoparietal channels^24^.

#### Single-sensor analyses with a priori EEG sensor

The *i*ERP method was applied with the CEEMDAN algorithm to EEG data from − 500 to 1200 ms relative to the stimulus onset to obtain the IMFs for each trial. The IF distribution of the IMFs (Fig. 2a, middle) suggested that the *i*ERP method could be applied using various partial sums from the second IMF (IF distributed mainly in the range of 15–32 Hz) to the last component. In the aforementioned example, the last component was formed by summing IMFs whose IFs were distributed below the delta band (i.e., summing from the sixth IMF to the representative “trend” IMF). These IMFs were denoted as IMF 6+. The final *i*ERP was baseline corrected relative to a 100-ms window prior to the stimulus onset^24^.

We applied the *i*ERP method for an a priori right temporoparietal channel^24^, namely “EEG065” (Fig. 2a, right), with TFCE repeated-measures analysis of variance (ANOVA) on the three conditions within the 2D [mode, time (50–700 ms)] domain (maximum *F* = 79.43, minimum *P* = 0.0002) (Fig. 2b). The yellow time windows in Fig. 2b indicate that *P* < 0.005 (0.05/10) for TFCE. Thus, in this statistical study, the MCP in the [mode, time] dimensions was resolved through TFCE. Moreover, the MCP was solved in the channel dimension if the number of interested electrodes (or electrode collections) for the cognitive task did not exceed 10. For comparison, we also used the conventional ERP method by applying a low-pass filter (Butterworth, order 5) with a cutoff frequency of 32 Hz to the averaged EEG data (Fig. 2c; with TFCE ANOVA of the three conditions in the time domain, *P* < 0.005). As expected, the results for mode 2:6+ were similar to those obtained with the conventional ERP method because these results were the partial sum of all the IMFs (including the trend IMF) whose IFs were below 32 Hz. The results for mode 2:6+ and the conventional ERP method revealed a negative deflection from 140 to 290 ms (N170). This deflection was greater for unscrambled faces than for scrambled faces. From approximately 315 ms until the end of the epoch, a slow negative potential shift differentiated the conditions. The *i*ERP method considered the importance of the P100 component across the 2:4, 2:5, 3:4, 3:5, 4:4, and 4:5 modes. The importance of the aforementioned component was ignored by the conventional ERP method and wideband 2:6+ mode. The face-related P100 effect has been demonstrated in other studies^25,26^. This effect is achieved through the filtering of ERPs by using an infrequent passband between 1 and 30 Hz^26^. The differences in the *i*ERPs among conditions were also identified from the statistical contrast between unscrambled faces (famous + unfamiliar) and scrambled faces and between famous and unfamiliar faces through the application of dependent-sample TFCE in the [mode, time] domain (*P*s < 0.025, as displayed in Fig. 2d,e). For comparison, the corresponding results obtained with the conventional ERP method are presented in Fig. 2f,g (TFCE in the time domain, *P*s < 0.025).

#### Multi-sensor analyses

**Figure 3:**
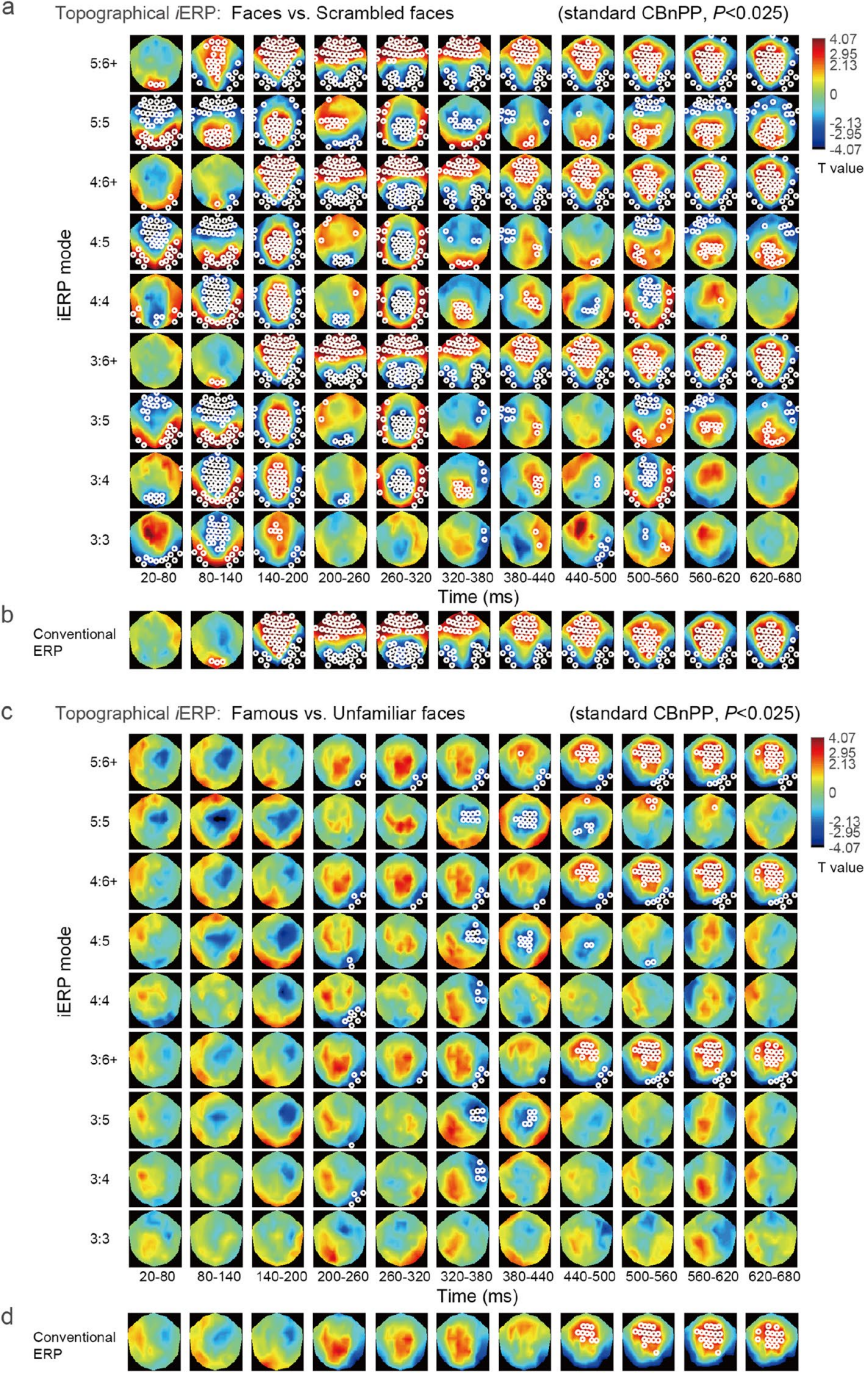
Topographical *i*ERP results. (**a**) Topographical contrast between the *i*ERP results for unscrambled faces (famous + unfamiliar) and scrambled faces. Statistical effects were identified using the standard CBnPP test in the 3D [channel, mode, time] domain. White circles denote the regions of “cluster *P*” < 0.025 in the testing domain. (**b**) Topographical contrast between the conventional ERP method results for unscrambled and scrambled faces. (**c**) Topographical contrast between the *i*ERP results for famous and unfamiliar faces. Conventions are the same as in (**a**). (**d**) Topographical contrast between the conventional ERP method results for famous and unfamiliar faces.

A topographical representation of the *i*ERP method that involves clustering experimental effects in the 3D [channel, mode, time] domain through the standard CBnPP test is presented (the maximum distance between channels was set as 40 mm for identifying neighbors). To ensure the accuracy of the representation, we averaged the *i*ERP from 20 to 680 ms prior to stimulus onset into 11 time bins of 60 ms each. Because the 60-ms bin size is longer than most periods of the second IMF (see the IF distribution of IMF2 in Fig. 2a), the *i*ERP modes initiated from the second IMF (i.e., mode 2: *k*, *k* = 2, …, 6+) could be neglected. We only emphasized modes that originated from the third-order IMF or higher. The topographical contrast of the *i*ERPs between unscrambled faces (famous + unfamiliar) and scrambled faces and between famous and unfamiliar faces is illustrated in Fig. 3a,c, respectively [5000 permutations, *α* = 0.025 (because we performed two comparisons here), two-sided]. The results of the wideband 3:6 + *i*ERP mode (Fig. 3a,c) were similar to those of the conventional ERP method (Fig. 3b,d, which correspond to the *i*ERP results in Fig. 3a,c, respectively). Both results clearly revealed the N170 effect in the contrast between unscrambled and scrambled faces (see the time bin at 140–200 ms). However, compared with the results of the conventional ERP method, the overall *i*ERP topographical results indicated additional effects for both of the aforementioned contrasts. First, a subtle but significant delay in the N170 effect was observed on temporoparietal channels for famous faces relative to unfamiliar faces at 200–260 ms. Second, *i*ERP modes 3:4, 3:5, 4:4, and 4:5 exhibited more negative deflection for famous faces than for unfamiliar faces on the electrodes at the right frontal brain area at 320–380 ms. This effect was not clearly observed in the results for the conventional ERP method or for *i*ERP modes 3:6+ and 4:6+. The aforementioned finding might indicate that when the low-frequency IMF 6+ mode is included in the partial sum, the right frontal negative effect, which is used to differentiate between famous and unfamiliar faces, is weakened.

#### Compact representations

**Figure 4:**
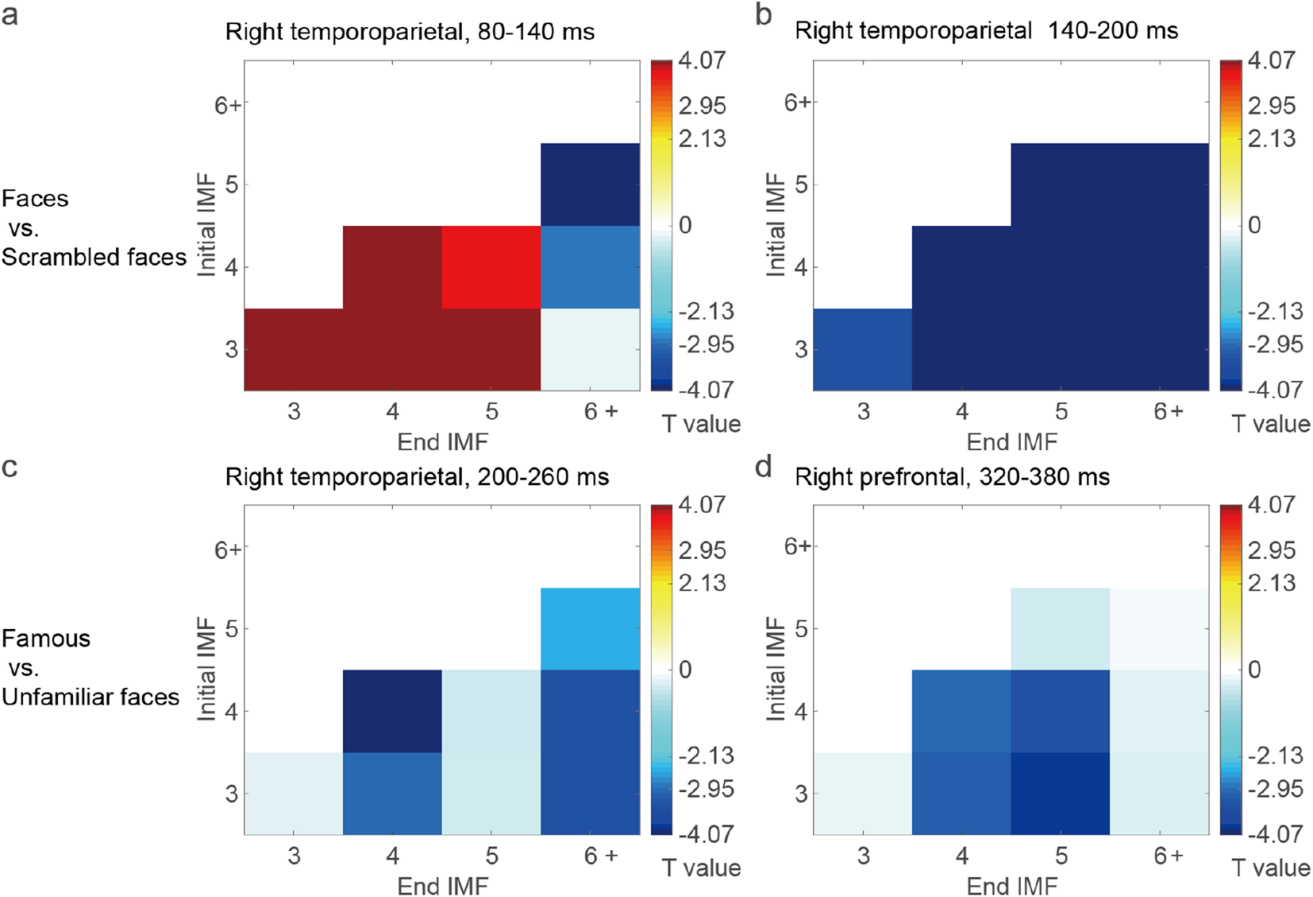
Compact representation of the *i*ERP results, where the *y*-axis and x-axis denote the initial and end IMFs of a mode, respectively. (**a**,**b**) For the contrast between unscrambled and scrambled faces, the right temporoparietal electrode cluster demonstrated clear P100 and N170 effects at 80–140 and 140–200 ms, respectively. However, the P100 effect was pronounced only if IMF 6+ was not included, whereas the N170 effect exhibited increased statistical effects in modes involving IMF 6+. (**c**) Compared with unfamiliar faces, famous faces exhibited more negative *i*ERP amplitudes in the right temporoparietal cluster at 200–260 ms. (**d**) A more negative deflection of the *i*ERP at the right prefrontal electrode cluster (EEG026, EEG027, EEG037, and EEG038) was observed for famous faces than for unfamiliar faces at 260–320 ms if a mode included IMF 4 but not IMF 6+.

For better visualization of the aforementioned topographical contrasts, a compact representation of the *i*ERP results in which the *y*-axis and *x*-axis respectively denote the initial and end IMF of a mode is displayed in Fig. 4. In this compact form, the *i*ERP effects in a specified electrode cluster are reorganized among the modes obtained from the aforementioned topographical analysis without the use of any further statistical tests. A compact representation of *i*ERP modes within a time bin was generated. For those in which the statistical effect of any channel within an electrode cluster was identified as significant in the previous topographical analysis, with the corresponding *P* value of the average *T* value of the electrode cluster below a certain threshold (0.025 in this study), the average *T* value of the mode is presented with completely opaque color. For the contrast between unscrambled and scrambled faces, the right temporoparietal electrode cluster (EEG003, EEG050, EEG060, and EEG065) demonstrated clear P100 and N170 effects in the time bins at 80–140 and 140–200 ms, respectively (Fig. 4a,b). However, the P100 effect was pronounced only if IMF 6+ was not included, whereas the N170 effect exhibited an increased statistical effect in modes involving IMF 6+. For the contrast between famous and unfamiliar faces, the right temporoparietal cluster revealed a negative effect in the time bin at 200–260 ms. This negative effect may have resulted from a delay in the N170 waveform for famous faces compared with that for unfamiliar faces (Fig. 4c). Furthermore, a more negative deflection was observed in the right prefrontal electrode cluster (EEG026, EEG027, EEG037, and EEG038) for famous faces than for unfamiliar faces in the time bin at 260–320 ms if a mode included IMF 4 but not IMF 6+ (Fig. 4d).

### *i*ERP for visual working memory

**Figure 5:**
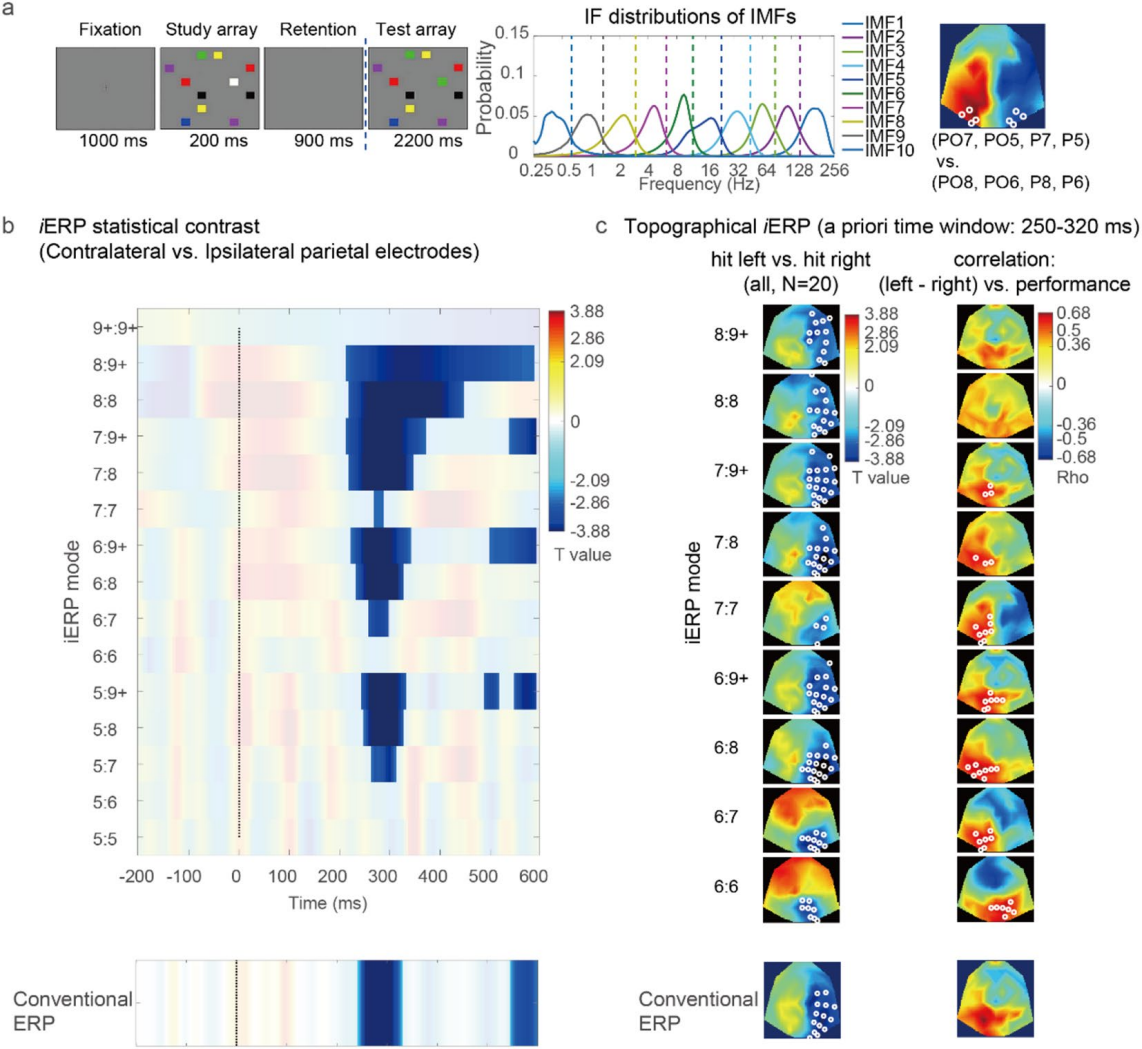
*i*ERP analysis on EEG data of the VWM task. (**a**) Illustration of the VWM task in the second example, IF distributions of the IMFs of the 1000-Hz EEG data, and parietal electrodes for determining the mean difference between the contralateral and ipsilateral amplitudes of the *i*ERPs. (**b**) Statistical contrast of the *i*ERPs between the contralateral and ipsilateral parietal electrodes, with the corresponding conventional ERP results shown below for comparison. The statistical results were obtained using a one-sample CBnPP test on the differences between the contralateral and ipsilateral amplitudes of the *i*ERPs within the 2D [mode, time (0–600 ms)] *i*ERP domain [for conventional ERP, the 1D (time) domain]. (**c**) Statistical contrast of the topographical *i*ERPs between the hit-left and hit-right conditions (first graph), which revealed a pronounced negative effect across all *i*ERP modes on the right parietal electrodes (trough *T* =  − 5.15, cluster *P* = 0.0016, *α* = 0.05, *N* = 20) but not a positive effect on the left parietal electrodes. The results in (**c**) are partially consistent with the expected pattern. Pearson correlation analysis revealed a pronounced positive correlation between the differential *i*ERPs (i.e., hit-left condition vs. hit-right condition) and the participants’ VWM capacity on the left parietal electrodes (peak *r* = 0.634, cluster *P* = 0.045, *α* = 0.05).

The second example demonstrates how to perform *i*ERP analysis on the basis of established ERP hypotheses. EEG data for this example were acquired from 20 participants when they performed a standard change detection task^27^ (Fig. 5a, left). These participants were required to memorize a study array comprising 11 colored rectangles and compare it with a subsequent test array (900 ms later) for determining whether the color of a rectangle in the left or right visual field changed. A preprocessing procedure was performed on the EEG data; thus, an artifact-rejected data set with a passband of 0.05–70 Hz and sampling rate of 1000 Hz was obtained^27^. This data set was originally used for investigating the ERP components of visual working memory (VWM) and the transcranial direct current stimulation effect on these components from the perspective of individual differences. Because the aforementioned data set was only used for demonstration purposes in the current example, we simply analyzed the *i*ERPs for a part of the original data set that involved only the sham condition (i.e., current fade in and out, with no current applied in between) and investigated its relationship with the VWM capacity (i.e., Pashler’s K)^28–30^.

To obtain the IMFs for each trial, *i*ERP analysis was performed using the CEEMDAN algorithm on EEG data from 800 ms prior to the study array onset to 600 ms after the test array onset. According to the IF distributions for the IMFs (Fig. 5a, middle), the *i*ERP modes were arranged using various partial sums from the fifth IMF (IFs distributed mainly in the range of 13–25 Hz) to the final component. In the current example, the final component (IMF 9+) was the sum of the IMFs with IFs below the delta range (i.e., from the ninth IMF to the “trend” IMF). The *i*ERP was baseline corrected relative to a 200-ms window prior to test array onset (this baseline window was often used in ERP research for attention and working memory^31,32^) to determine the *i*ERP effects during VWM retrieval and comparison.

#### Contralateral-sensor analyses

For this task, an established ERP hypothesis is that the ERP effects related to visual attention or working memory are influenced by the mean difference between the contralateral and ipsilateral amplitudes at parietal electrodes relative to the target side [e.g., N2pc, and sustained parietal contralateral negativity (SPCN)]. A priori time windows, such as the selected window from 250 to 320 ms for analyzing N2pc^27^, were selected for investigating these ERP effects. According to the aforementioned hypotheses, we first analyzed the mean difference between the contralateral and ipsilateral amplitudes of *i*ERP at the parietal electrodes [(PO7, PO5, P7, P5) vs. (PO8, PO6, P8, P6)] (Fig. 5a, right) relative to the side of the correctly detected changed item. Figure 5b displays the statistical contrast between the *i*ERP results for the contralateral and ipsilateral parietal electrodes. The corresponding contrast of the conventional ERP results is also shown at the bottom of Fig. 5b for comparison. The aforementioned contrasts were achieved through a one-sample CBnPP test of the differences between the contralateral and ipsilateral amplitudes of the *i*ERP within the 2D [mode, time (0–600 ms)] *i*ERP domain (for the conventional ERP method, only in the 1D “time” domain). The results of the CBnPP test indicated that the N2pc effect was the most stable effect across almost all modes except those derived from a single IMF (i.e., modes 5:5, 6:6, and 7:7) and mode 5:6. Compared with the N2pc effect, the SPCN effect was weaker in the *i*ERP and conventional ERP methods. However, the *i*ERP contrast revealed a prolonged negative effect in the low-frequency mode 8:9+ from 220 to 570 ms and a short positive marginal effect (cluster *P* = 0.075) in the modes 7:7 and 6:7 from 350 to 450 ms. The aforementioned results might explain why the prolonged negative effect could no longer be observed when modes 7:9+, and 6:9+ were summed. Thus, the *i*ERP method might have separated these originally overlapping components into their corresponding modes.

#### Multi-sensor analyses with a priori time window

We analyzed the topographical *i*ERP of the aforementioned task for an a priori time window of 250–320 ms for the N2pc effect. Thus, topographical *i*ERP analysis was performed according to the statistical contrast between the correctly detected left and right change trials within the 2D [channel, mode] *i*ERP domain (one-sample CBnPP test ,the maximum distance between channels was set as 40 mm for identifying neighbors). An antisymmetric pattern with a negative effect on the right parietal electrodes and positive effect on the left parietal electrodes was expected. In Fig. 5c, every topographical *i*ERP result is displayed with its corresponding conventional ERP result for comparison. The first graph in Fig. 5c illustrates the statistical contrast of the topographical *i*ERPs between the hit-left and hit-right conditions (*N* = 20). The aforementioned results revealed a pronounced negative effect across all *i*ERP modes on the right parietal electrodes (trough *T* =  − 5.15, cluster *P* = 0.0016, *α* = 0.05) but not a positive effect on the left parietal electrodes. Thus, the results were partially consistent with the expected pattern. However, a pronounced positive correlation was observed between the differential *i*ERPs (i.e., hit-left condition vs. hit-right condition) and the participants’ VWM capacity for the left parietal electrodes (peak *r* = 0.634, cluster *P* = 0.045, *α* = 0.05). This positive correlation indicated that the higher the participants’ VWM capacity, the more their hit-left versus hit-right contrast of *i*ERPs was consistent with the expected antisymmetric topographical pattern (Fig. 5c, second graph).

## Discussion

In this study, we propose the *i*ERP analysis method and demonstrate its usage and advantages by using two examples. Compared with the conventional ERP method, the *i*ERP method can better resolve ERP components in their characteristic time scales. In addition, in contrast to the event-related mode analysis method, the *i*ERP method can avoid a trial-and-error process for determining the relevant IMF or partial sum of IMFs for each studied ERP component.

**Figure 6:**
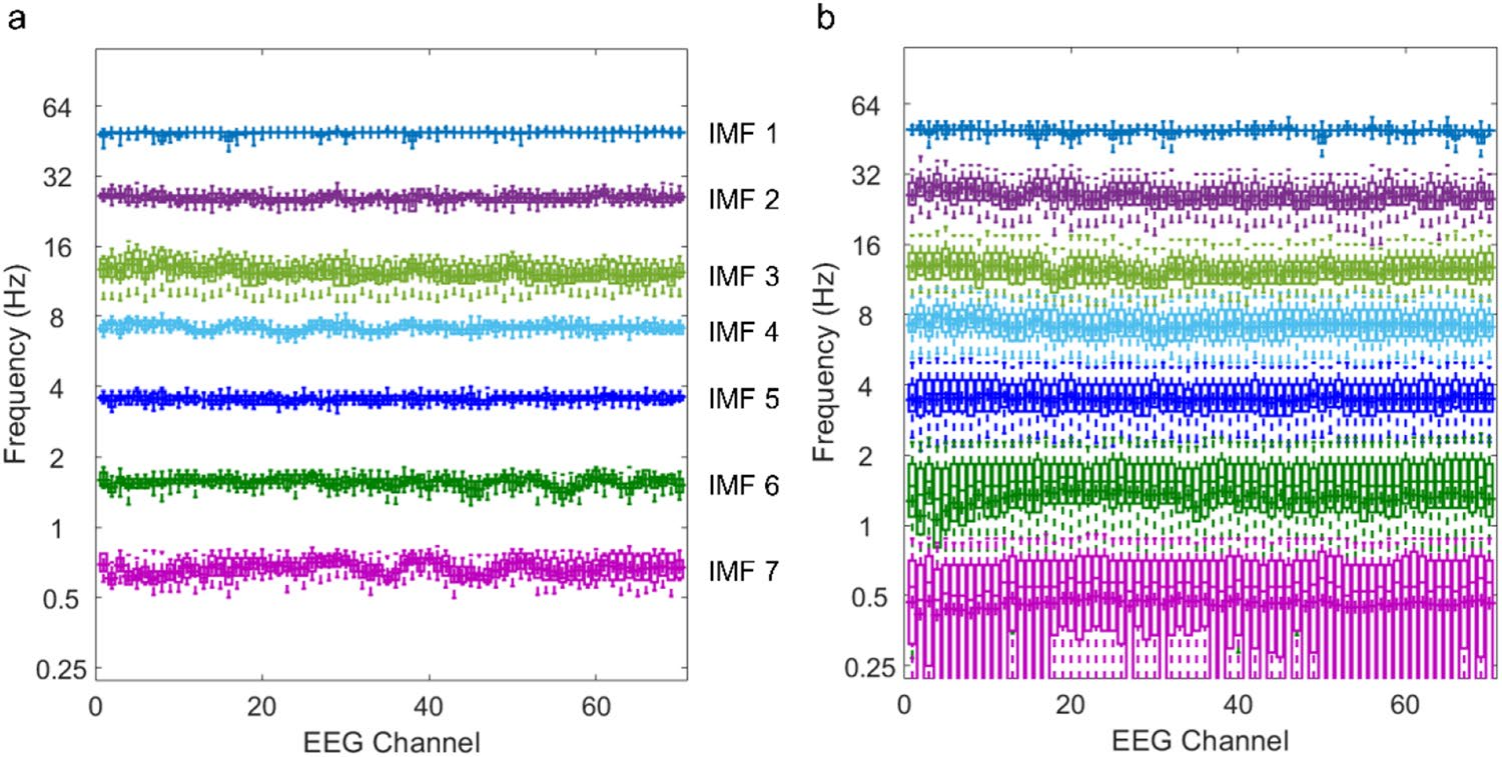
Time scale consistency in the order of IMFs investigated through the box-and-whisker plots (the box depicting the median and the 25th and 75th quartiles and the whisker showing the 5th and 95th percentile) of instantaneous frequencies of IMFs of EEG data in the first example. (**a**) Between-subjects analysis: interchannel and intersubject time scale consistency in the order of IMFs. Each box-and-whisker plot represents the distribution of subjects’ representative frequencies of a specific IMF order at a given channel. For each subject, the representative frequency of a specific IMF order at a given channel is the frequency with the maximum occurrence probability in the instantaneous frequency distribution of IMFs of the order at the given channel. (**b**) Within-subject analysis (only for subject 10): interchannel and intertrial time scale consistency in the order of IMFs. Each box-and-whisker plot represents the distribution of instantaneous frequencies of IMFs of a specific order at a given channel from all trials.

The CEEMDAN algorithm used in this study is based on the 2014 improvement of the CEEMDAN algorithm proposed by Colominas et al.^14^. Their improved CEEMDAN algorithm overcomes the problems of residual noise and spurious modes that were observed in its earlier version^16^ and can thus be used to generate IMFs with high resistance to the mode-mixing phenomenon and noise residuals. Moreover, we incorporated complementary noise pairs into the CEEMDAN algorithm to further reduce the noise residual and improve the interchannel, intertrial, and intersubject time scale consistency of the *order of IMFs* according to the requirements of the proposed method. This high interchannel, intertrial, and intersubject time scale consistency in the order of IMFs can be investigated through the EEG data of the first adopted example in this study by looking into the between-subject (Fig. 6a) and within-subject (Fig. 6b) distribution of instantaneous frequencies for each order of IMFs at each channel. Although we cannot completely rule out the possibility of time scale inconsistency in the order of IMFs across channels, trials, and subjects in any other data sets that we have not investigated, however, this inconsistency can hardly be found in the two adopted examples in this study and in multiple EEG data sets that we have explored, as long as the data are acquired and preprocessed with the same procedure. Therefore, for the proposed *i*ERP analysis, at this moment we are not inclined to offer a solution to deal with this rarely occurred inconsistency, though it is conceivable that a possible solution may be to correct the order of IMFs by comparing their frequency distributions.

Because of the high intertrial and intersubject time scale consistency in the order of IMFs, the characteristic time scale of an *i*ERP mode is closely related to the order of its constituent IMFs, and this time scale will be consistent across subjects. For a pure *i*ERP mode, it is obvious that its time scale will be similar to the characteristic time scale of the corresponding IMF in each trial that constitutes the mode. However, for a composite *i*ERP mode, since it is underpinned by its corresponding partial sum of IMFs in each trial, its time scale can only be estimated after the mode has been formed (similar to the approach used for an IMF, measured by the time lapse between the extrema). For a cognitive task, a critical *composite i*ERP mode may be derived from the nonlinear characteristics of the ERP component represented by the mode. This is based on the fact that one of the characteristics of a nonlinear oscillation system is that its dynamics can be expressed by an oscillation at its fundamental frequency, together with a series of harmonics^33^ (multiples of the fundamental frequency). Thus, when the degree of nonlinearity of an oscillation system goes high such that it cannot be revealed merely by the *variation of instantaneous frequency* within a single IMF (which is also a character of nonlinearity)^34^, two or more IMFs are required to represent the dynamics of the nonlinear system.

We note that the *i*ERP analysis is not a method of decomposition, but a method of representation for ERP components. The meaning of a *pure i*ERP mode (e.g., mode 4:4 or 6:6) is quite simple: the mean waveform of brain activities within a particular frequency band. Because of the natural-filtering property of the CEEMDAN (inherited from EMD), the mean waveform can reveal some nonlinear characteristics on the waveform shape. Suppose a pure *i*ERP mode has a higher amplitude in one experimental condition as compared to the other experimental condition, the next step could be using Hilbert-Huang Transform^2^ (HHT) to see whether the spectral power of the condition is also higher than the other condition. If it is not, the following step could be considering whether the condition has higher inter-trial coherence (ITC), which can be estimated by computing the inter-trial phase clustering^35^ (ITPC) with instantaneous phases of the corresponding IMF in each trial. If it is, then we can conclude that the effect in the pure iERP mode resulted from higher phase consistency across trials. In contrast to pure *i*ERP modes, the meaning of a composite *i*ERP mode could be more complicated. In addition to the abovementioned issue of the high degree of nonlinearity, it can also be derived by a mechanism of cross-frequency phase-phase coupling^36,37^ in which instantaneous phases of one IMF couples with instantaneous phases of the other IMF in an n:m ratio. In the dyadic structure of IMFs, the phase-phase coupling may primarily occur in the 1:2, 1:4, and 1:8 ratios. Thus, when one of these coupled oscillations is also phase-locking to a stimulus (i.e., inter-trial coherence) in an experiment, the entire mechanism of phase couplings contributes to a pronounced composite *i*ERP mode.

In the proposed method, because the CEEMDAN (as well as EMD and EEMD) is a nonlinear process, different from conventional Fourier-based linear filters (e.g., FIR or IIR filters), the order of its application is crucial: the CEEMDAN should be performed on each individual trial rather than on averaged data. Therefore, in the *i*ERP analysis, the process of averaging over trials for an IMF or a partial sum of IMFs can be executed only when the IMFs for each trial have been obtained.

The *i*ERP results obtained in the two adopted examples suggest that clustering statistical effects in the *i*ERP 2D [mode, time] or 3D [channel, mode, time] domain may enhance the sensitivity of expected effects. Such enhancement may be achieved because an ERP effect might be best revealed in a certain *i*ERP mode. The total effect within a cluster, obtained through the combination of the effects from all neighboring modes, demonstrated high sensitivity of the expected effect. Moreover, research has indicated that ERP components typically overlap with their adjacent components in time and space^38^, which might cause mutual interference among these components, thus limiting the statistical effect from an individual ERP component. The proposed method may be able to resolve this “component overlap” problem when temporally adjacent components can be characterized by different time scales, as displayed in Fig. 5b. Furthermore, each of the two cluster-based statistical methods (i.e., CBnPP and TFCE) demonstrated in the two adopted examples, by the algorithm itself, can avoid the problem of overfitting when an addition dimension (i.e., “mode”) is added to the space of testing data. This originates from the fact that this kind of cluster-based statistical methods is achieved by testing the cluster-level observed test statistics (i.e., without permutation) against the cluster-level random test statistics (i.e., the permutation distribution). Therefore, when the clusters of observed test statistics are formed based on the higher-dimensional *i*ERP neighbor relationship and get greater observed statistical values (by taking the sum over each cluster), the cluster-level random test statistics are also obtained on the basis of the higher-dimensional neighbor relationship and have larger random statistical values. To verify that the *i*ERP analysis is not enhancing the sensitivity of distinguishing different experimental conditions at the cost of increasing the possibility of identifying an actually null effect as significant, we perform the *i*ERP analysis on a simulated data set in which two conditions should be no different. This data set is generated by acquiring the first 30 trials of “unfamiliar faces” from each participant’s EEG data at a temporoparietal channel in the first adopted example in this study first. For each participant, the next 30 trials, serving as a “pseudo-condition”, are produced by adding white Gaussian noise (WGN) to the first 30 trials with the signal-to-noise ratio equal to 4. The statistical contrast of *i*ERP between the unfamiliar and the pseudo conditions identified by the dependent-sample TFCE in the [mode, time] domain does not show any significant effect (*N* = 16, minimum *P* = 0.664), as shown in Fig. S1a (see Supplementary Figure S1). This is further verified by a cross-validation analysis in which the above TFCE test is performed on 100 sub-datasets of the simulated data individually, each formed by randomly choosing 14 participants from the original 16 participants (i.e., *N* = 14). Fig. S1b shows that the 100 minimum *P* values of the cross-validation analysis are distributed from 0.15 to 1 (*P*s ≫ 0.05), suggesting that the *i*ERP analysis will not introduce an additional probability of false alarm and overfitting.

In summary, this study proposes a new approach, namely the *i*ERP method, for investigating ERP components by introducing an additional “mode” dimension for ERP representation. Incorporating a special definition of neighbors for every mode enables adequate clustering of the statistical effects of *i*ERPs, thus preventing the additional dimension from reducing the sensitivity of expected effects. By contrast, the *i*ERP method enhances sensitivity in most cases, as indicated by the results obtained for the two examples. Therefore, we recommend that all previously presented ERP data, especially those that originally demonstrated “no effect” in their expected ERP components, can be re-examined using the proposed approach.

## Supplementary Information

Supplementary Figure S1.

## Data Availability

A minimal set of data and custom MATLAB codes that can be used for replicating main results in the two adopted examples of this work are available from the corresponding project in the public repository: https://osf.io/2pnd9/(DOI:10.17605/OSF.IO/2PND9). These data and codes are open access under the GNU general public license (GPL) v2.0.

## Code availability

In the aforementioned public repository, the subfolder “ierp_tools_d1” contains a set of necessary functions (including an installation guide and an installing script: “install_ierp.m”) for performing iERP analysis as well as their wrappers for analyzing data in SPM12 format (http://fil.ion.ucl.ac.uk/spm/). If functions in "ierp_tools_d1" are correctly installed, by following the steps described on the “Home” of the project, readers will be able to reproduce results of the two examples and thus apply the proposed method to their own study.
