# Supplementary material for: Event-related components are structurally represented by intrinsic event-related potentials
Source: Sci Rep. 2021 Mar 11;11:5670. doi: 10.1038/s41598-021-85235-0 (PMC7970958; doi:10.1038/s41598-021-85235-0)
Supplement: Supplementary file 1 — Supplementary Figure S1. [file 41598_2021_85235_MOESM1_ESM.pdf]

Supplementary Material for

Event-related components are structurally represented by intrinsic event-related potentials

Chong-Chih Tsai, Wei-Kuang Liang*

Wei-Kuang Liang
Email: wkliang@cc.ncu.edu.tw

This file includes:

Figure S1

a

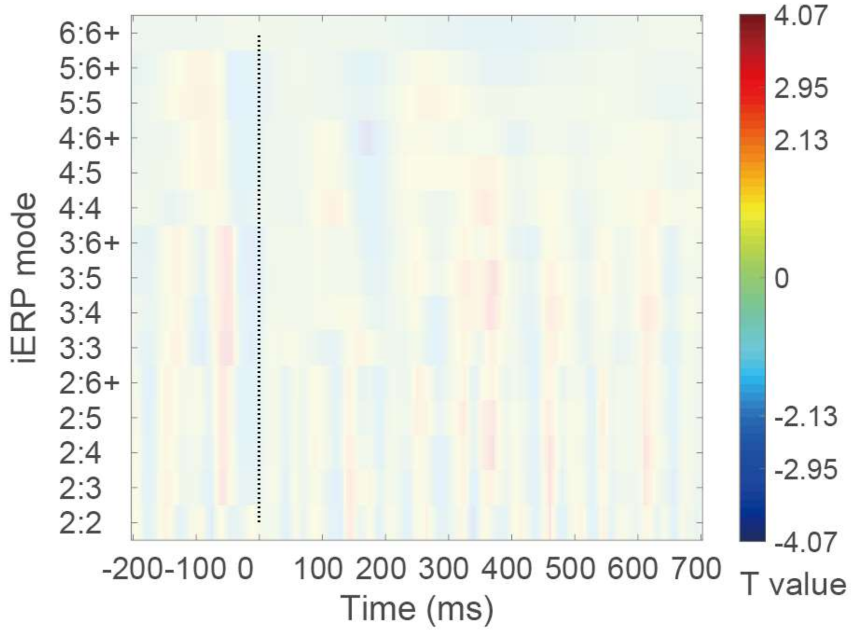

b

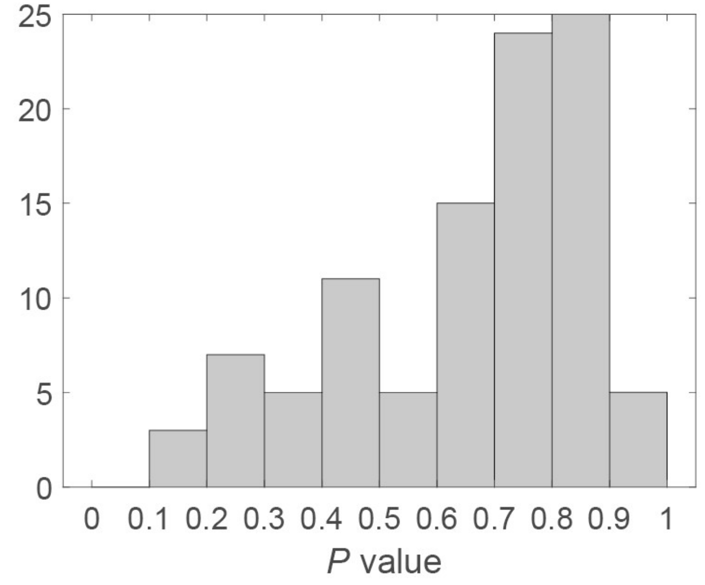

Fig. S1. To verify that the *i*ERP analysis is not enhancing the sensitivity of distinguishing different experimental conditions at the cost of increasing the possibility of identifying a null effect as significant, we perform the *i*ERP analysis on a simulated data set in which two conditions should be no different. This data set is generated by acquiring the first 30 trials of "unfamiliar faces" from each participant's EEG data at a temporoparietal channel in the first adopted example in this study first. For each participant, the next 30 trials, serving as a "pseudo-condition", are produced by adding white Gaussian noise (WGN) to the first 30 trials with the signal-to-noise ratio equal to 4. (a) The statistical contrast of *i*ERP between the unfamiliar and the pseudo conditions identified by the dependent-sample TFCE in the [mode,

time] domain (*N*=16, minimum *P*=0.664). (b) Distribution of 100 minimum *P* values of a cross-validation analysis in which the above TFCE test is performed on 100 sub-datasets of the simulated data individually, each formed by randomly choosing 14 participants from the original 16 participants (i.e., *N*=14). Both results support that the *i*ERP analysis will not introduce an additional probability of false alarm and overfitting.
